# Incidental Diagnosis of Cyclic Thrombocytopenia: An Asymptomatic Case

**DOI:** 10.7759/cureus.66310

**Published:** 2024-08-06

**Authors:** Fatma Keklik Karadağ, Ajda Gunes, Nihal Mete Gokmen, Fahri Şahin, Guray Saydam

**Affiliations:** 1 Department of Hematology, Izmir City Hospital, Izmir, TUR; 2 Department of Internal Medicine, Division of Hematology, Ege University Faculty of Medicine, Izmir, TUR; 3 Department of Internal Medicine, Division of Allergy and Immunology, Ege University Faculty of Medicine, Izmir, TUR

**Keywords:** cytopenia, episodic, fluctuation, immune thrombocytopenia, cyclic thrombocytopenia

## Abstract

Cyclic thrombocytopenia (CTP) is a very rare condition that is characterized by episodic thrombocytopenia over a period of three to five weeks. The pathogenesis of CTP is unclear and most likely heterogeneous; however, usually there is no clue about the underlying disease. In this case report, we presented a 48-year-old female who had a low platelet count of 66 x 10^3^/µL (range: 150-450 x 10^3^/µL) on her routine examination with no evidence of bleeding. On further review of her laboratory workup in the past several years, she was noted to have multiple episodes of low platelet counts. She was diagnosed with CTP after a recurrent pattern of fluctuations in her platelet count, with improvements sometimes without intervention. Unfortunately, most CTP patients are misdiagnosed as having primary immune thrombocytopenia (ITP), and CTP typically responds poorly to ITP therapy. This case underscores the need for further research and serves as a valuable reference to increase the awareness of clinicians about CTP.

## Introduction

Cyclic thrombocytopenia (CTP) is a very rare disease that is characterized by periodic fluctuations in peripheral platelet counts from very low levels to normal or above normal levels. These oscillations have been observed with periods varying between three and five weeks. However, only platelet fluctuation is expected in CTP, and a concomitant presentation of CTP with neutropenia could be seen [1,2]. Besides the fact that the underlying disorder is unknown in most cases, CTP related to hematologic diseases such as myeloid neoplasms, thyroid gland disorders, menstrual cycles, and viral infections has been described so far. Females are affected more than males by the menstrual period [2]. Nadir platelet counts could be variable, between 1 and 90 × 10^3^/µL, with or without bleeding tendency [3]. Typically, mild or moderate mucocutaneous bleeding is expected when the platelet count is at its lowest point. Currently, there are no guidelines for the diagnosis and management of CTP due to its rarity and the unawareness of physicians. This case report examines a 48-year-old female with CTP, detailing her clinical presentation, diagnostic procedures, and treatment approaches, including a surgery procedure. The report aims to contribute to existing literature and stimulate further research, complemented by an extensive literature review.

## Case presentation

A 48-year-old female was referred to Ege University's hematology department in September 2021 after she had been found to be thrombocytopenic with a platelet count of 66 x 10^3^/µL at her preoperative evaluation. The patient's past and family medical history were unremarkable for his bleeding tendency. She has a past surgical history of total thyroidectomy for thyroidal papillary microcarcinoma, and she was on thyroid hormone replacement therapy. On admission, a physical examination showed a few petechiae on the lower extremities, and there was no lymphadenopathy or hepatosplenomegaly. The white blood cell count was 7.71 x 10^3^/µL (range: 4.5-11 x 10^3^/µL), the neutrophil count was 4.11 x 10^3^/µL with a normal range, and the hemoglobin level was 14.3 g/dL (range: 11.7-16 g/dL). The peripheral blood smear was normal, other than decreased platelets with no clumping. Coagulation studies showed no abnormality. Additionally, her viral serology (i.e., HIV, hepatitis B and C), vitamin levels (i.e., vitamin B12 and folate), lactic dehydrogenase, and iron studies were normal. Antinuclear antibody screening, lupus anticoagulant, and rheumatoid factor were negative. Bone marrow examination revealed normal cellularity and morphology of all kinds of cells and no infiltration of malignancy. The platelet count increased to 92 x 10^3^/µL spontaneously seven days later. On further review of laboratory workup in the past several years, she was noted to have multiple episodes of low and normal platelet counts ranging from 18 to 220 x 10^3^/µL since 2015. She was recommended to check her platelet count every week for at least eight weeks; however, she did not want to postpone the breast biopsy. She was given a unit of platelets before the surgery, and her platelet level increased from 66 to 122 x 10^3^/µL. The breast reduction surgery was performed successfully.

**Figure 1:**
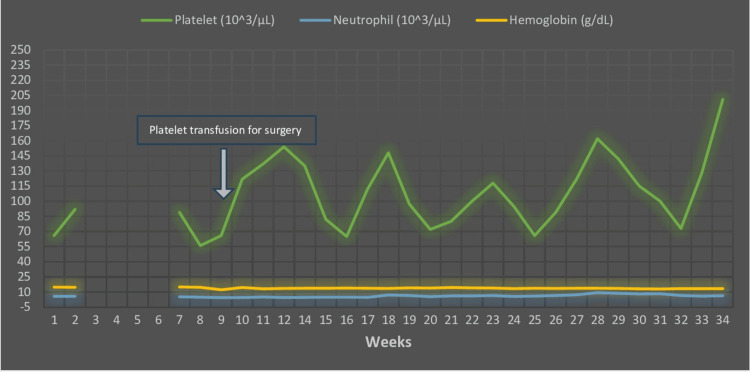
Change in platelet, neutrophil, and hemoglobin count during the follow-up The platelet counts fluctuated (ranging from 56 to 201 x 10^3^/µL) with each cycle. There were four platelet cycles at about 35-day intervals.

After a while from the surgery, upon close observation of her platelet count, we found oscillations in the platelet count, ranging from 56 to 201 x 10^3^/µL with a cycle of 28-38 days (Figure 1). We diagnosed her condition as CTP. She has undergone routine examinations every two to three months, including a total blood cell count and peripheral blood smear, in our outpatient clinic since December 2021. Even though she had severe thrombocytopenia (in September 2018 and July 2020; 18 x 10^3^/µL and 25 x 10^3^/µL, respectively) previously, her platelet counts did not fall below 50 x 10^3^/µL without any treatment during our follow-up period.

## Discussion

The incidence of CTP is notably low, with a documented count of fewer than a hundred cases in the existing literature. Only 4 (0.7%) of 614 patients who were evaluated for thrombocytopenia (<150 × 10^3^/µL) were diagnosed with CTP in a tertiary hematology center in Canada [4]. According to Go's systematic review, the presenting symptoms are usually related to mild to moderate bleeding episodes, even though a few asymptomatic CTP patients have been reported [3]. Patients affected by CTP experience recurring thrombocytopenia with or without symptoms every three to five weeks that resolve spontaneously in a few days. Our case is asymptomatic, and thrombocytopenia was found incidentally just before the surgical procedure. In her previous laboratory results, a spontaneous increase to normal levels from a very low platelet count was remarkable for CTP. The platelet count was monitored weekly for almost three months. After the observation of periodic platelet count fluctuations every 28-38 days, the diagnosis of CTP was established. Notably, our case exhibited unique features not commonly reported in the extant literature, such as the absence of bleeding episodes.

There are some well-described situations in CTP: (a) the half-life of the circulating platelets is short; (b) megakaryocytes appear normal in the bone marrow; and (c) cyclic oscillation of megakaryocytes in parallel with platelet counts [3]. However, the exact pathogenetic mechanism of CTP is still unknown. Cyclic autoimmune destruction of platelets and cyclic fluctuations of platelet production due to infections, hormonal imbalance during the menstrual period, and associated other diseases are supposed to be the reasons for CTP [5-7]. The relationship between autoimmune thyroid disease and ITP has been well described previously. Nevertheless, thyroid gland diseases are reported commonly in patients with CTP, especially in female patients [8]. As noted, our case had a disorder of the thyroid gland too.

It is well known that most CTP patients are often misdiagnosed and treated as having primary immune thrombocytopenia (ITP). In contrast to ITP, transient thrombocytopenia in CTP is resistant to ITP treatment options such as corticosteroid, immunoglobulin, splenectomy, immunosuppressive, or thrombopoietin receptor agonists [9]. Some patients who were successfully treated with cyclosporine A, azathioprine, danazol, hormonal contraception, and thyroxine have been reported so far; however, there are no exactly recommended treatment and follow-up guidelines for CTP yet. Spontaneous remissions may occur in CTP, even after many years from diagnosis [9,10]. Some of the patients with CTP do not require any medical intervention, like in our case. If the fluctuation of platelets had not been recognized, our patient would most likely have been treated with steroids or immunoglobulin before the surgery. After the diagnosis, the follow-up of these patients depends on clinical findings. We believe that for patients without any bleeding episodes, a follow-up every three to six weeks at the beginning and then extending for two to three months would be reasonable according to the fluctuation of platelet count; however, weekly monitoring would be required for those with severe thrombocytopenia and bleeding episodes.

## Conclusions

The diagnosis of CTP is based on the periodic oscillation of platelets with significant amplitude. This case contributes to the limited body of literature on CTP by highlighting unique features such as the absence of bleeding history. Currently, no treatment is effective for every patient with CTP. Treatment of underlying disease (i.e., low-dose hormonal contraception, thyroxine, Helicobacter pylori eradication, anti-viral therapy for hepatitis, etc.) and immunosuppressive agents such as cyclosporine A and azathioprine have been reported so far. However, some of the cases do not need any therapy, and spontaneous remission can occur. Our case underscores the early recognition that CTP can prevent the patient from potentially harmful and extended therapies. These insights could serve as valuable reference points for clinicians and researchers in the field.

## References

[1] Langlois GP, Arnold DM, Potts J, Leber B, Dale DC, Mackey MC (2018). Cyclic thrombocytopenia with statistically significant neutrophil oscillations. Clin Case Rep.

[2] Şumnu A, Diz-Küçükkaya R (2010). Cyclic thrombocytopenia: a case report. Turk J Haematol.

[3] Go RS (2005). Idiopathic cyclic thrombocytopenia. Blood Rev.

[4] Arnold DM, Nazy I, Clare R, Jaffer AM, Aubie B, Li N, Kelton JG (2017). Misdiagnosis of primary immune thrombocytopenia and frequency of bleeding: lessons from the McMaster ITP Registry. Blood Adv.

[5] Furuyama H, Koga Y, Hamasaki K, Kuroki F, Itami N, Ishikawa Y (1999). Effective treatment of cyclic thrombocytopenia with cepharanthin. Pediatr Int.

[6] Rocha R, Horstman L, Ahn YS, Mylvaganam R, Harrington WJ (1991). Danazol therapy for cyclic thrombocytopenia. Am J Hematol.

[7] Dan K, Inokuchi K, An E, Nomura T (1991). Cell-mediated cyclic thrombocytopenia treated with azathioprine. Br J Haematol.

[8] Steinbrecher O, Mitrovic M, Eischer L, Šinkovec H, Eichinger S, Kyrle PA (2020). Clinical and laboratory characteristics of cyclic thrombocytopenia: an observational study. Haematologica.

[9] Kyrle PA, Eichinger S (2021). How I manage cyclic thrombocytopenia. Blood.

[10] Zhang H, Villar-Prados A, Bussel JB, Zehnder JL (2024). The highs and lows of cyclic thrombocytopenia. Br J Haematol.

